# Monitoring serologic response to single *in ovo* vaccination with an immune complex vaccine against infectious bursal disease in broilers

**DOI:** 10.1016/j.psj.2021.01.022

**Published:** 2021-01-19

**Authors:** C. García, J.M. Soriano, V. Cortés, S. Sevilla-Navarro, C. Marin, J.L. Balaguer, P. Catalá-Gregori

**Affiliations:** ∗ Center for Poultry Quality and Animal Feeding of the Valencian Community (CECAV), Castellón, Spain; † Food & Health Lab, Institute of Materials Science, University of Valencia, 46980 Paterna, Valencia, Spain; ‡ Departamento de Producción y Sanidad Animal, Salud Pública Veterinaria y Ciencia y Tecnología de los Alimentos, Instituto de Ciencias Biomédicas, Facultad de Veterinaria, Universidad Cardenal Herrera-CEU, CEU Universities, 46113 Moncada, Spain; § Department of Animal Production and Health, Veterinary Public Health and Food Science and Technology, Institute of Biomedical Sciences, Faculty of Veterinary Medicine, Cardenal Herrera-CEU University, CEU Universities, Moncada, Spain

**Keywords:** ELISA, GeoServer, IBD, broiler, vaccination

## Abstract

The infectious bursal disease (**IBD**) virus is one of the most resistant and prevalent virus worldwide in the poultry industry, being vaccination the main tool to control the disease. For this reason, consistent and uniform immunization of broiler flocks against IBD is necessary to avoid the disease spreading. The aim of this study was to apply and assess an epidemiologic mapping tool focused on the immunization by in ovo single broiler vaccination using an immune complex IBD vaccine. With this regard, 7,576 serum samples were collected from 603 broiler flocks raised in 354 Spanish farms. To do so, blood samples were randomly collected from birds with ages between 35 to 51 d, and the serum was analyzed by ELISA. The results obtained from this study suggested a high uniform immunization against IBDV and a protective immunization between 35 and 51 d of age, with mean titer values ranging between 6,331 and 7,426. In addition, seroprevalence titer data of this large-scale monitoring study fitted a polynomial equation with a R^2^ value of 0.77, helping to explain and predict the humoral response to IBD vaccination. This seroprevalence map was applied to broiler production and was based on business intelligence tool that incorporates newly developed mapping tool to cover the need of having real-time information of humoral response to IBD vaccination and could be an effective tool for veterinary services to control and prevent IBD.

## Introduction

Infection bursal disease (**IBD**) is an acute and highly contagious viral infection in birds that has lymphoid tissue as its primary target, with a particular predilection for the bursa of Fabricious (cloacal bursa), which mainly affects chickens. The first cases were observed in the area of Gumboro (Delaware, USA), so that in 1966 became known as Gumboro disease (Van den Berg et al., 2000).

The IBD virus (**IBDV**) is a member of the family Birnaviridae and genus *Avibirnavirus* (Dobos et al., 1979; Müller et al., 1979). The IBDV is stable under extreme environmental conditions and resistant against several disinfectants treatments (Benton et al., 1967; Shirai et al., 1994). Poultry facilities still infective for a long period after depopulation, including water, feed, droppings, and especially faeces, remain contaminated for several wk (Zhao et al., 2013). Besides, it has been described that pests, such as lesser mealworm (*Alphitobius diaperinus*) and mosquitoes (*Aedes vexans*), could act as reservoirs of the IBDV (Skeeles et al., 1979; Howie and Thorsen, 1981). The hardy nature of this virus is one reason for its persistent survival in poultry houses (Eterradossi and Saif, 2008).

Currently, vaccination is the main measure to control IBD at field level. Different vaccines such as conventional live attenuated vaccines in drinking water, vector-based vaccines subcutaneous or in ovo, and immune complex vaccines subcutaneous or in ovo are described to be used in the poultry sector. Regarding the classic vaccines used, they are applied via drinking water and are based on classical virulent strains, which can induce moderate-to-severe bursal lesions and even immunosuppression. To avoid unwanted effects, the poultry sector has developed less-aggressive and beneficial vaccines for birds, reducing adverse reactions and improving their handling. In ovo vaccination causes better development of the immune system and minimizes the risks of contamination and disease spreading. Moreover, the vaccination injection is uniform, precise, and reduces birds handling and therefore the stress produced by this practice (Müller et al., 2012).

In young chicks, normally variable, but high levels of maternal antibodies, persist that in most cases prevents the traditional live vaccines from inducing active protection after single or even repeated applications. The recently developed IBDV immune complex vaccine prepared by combining live vaccine virus with a specific antibody to the IBDV (Whitfill et al., 1994) may be applied in ovo or at day-old. A great advantage of this type of vaccine is the start of the replication. With regard of the maternal antibody level, the vaccine virus strain starts to replicate at the most appropriate time to provide active immune response to the animals (Sharma et al., 1985; Haddad et al., 2000; Kelemen et al., 2000).

The proper immunization of the animals is monitored by ELISA (Babatunde et al., 2018). This easy and cost-effective tool has been used previously to develop maps of seroprevalence to control different poultry diseases, such as avian influenza, *Mycoplasma gallisepticum,* or *Salmonella* (Garcia et al., 2015, 2016, 2018). The fact of using the spatial and temporal distribution of a disease over time with seroprevalence maps can allow the technicians of the sector to anticipate any dangerous situation, as well as to make decisions quickly before the onset of control loss of a major disease.

To the best of our knowledge, current OIE mapping tools in IBD monitoring lack the following (World Animal Health Information Database (WAHIS Interface), 2012): do not include IBD seroprevalence data, only include reported outbreaks and/or disease; are not in real time (concerning disease distribution mapping, the period shown is 6 mo equivalent to 3 broiler complete flock rearing period); do not include lower geographical regions; and do not include production types as “broilers.”

In this context, the aim of this study was to apply and assess a mapping epidemiologic tool to control IBD by the knowledge of the immunization by in ovo single broiler vaccination using an immune complex IBD vaccine.

## Materials and method

Vaccination was performed in the hatchery at 18-day-old chicken embryos with a single in ovo immune complex vaccine against IBD (CEVAC TRANSMUNE, Ceva Santé Animale, France).

### Sample Collection

During this study, a total of 603 flocks from 354 Spanish broiler farms were sampled. From each farm, 10 to 15 blood samples were collected from animals older than 35 d of age to avoid maternal antibodies. To this end, venipuncture was performed with a needle or scalpel in the brachial vein, and the blood was collected in a 5-mL glass tube (1 tube per animal) to obtain about 3 mL of sample. The tubes were kept horizontally at the room temperature until clot formation and then subsequently cooled until arrival at the laboratory.

### Sample Analysis

Once in the laboratory, all samples were recorded using the program ORALIMS (Nobel Biocare AB, Gothenburg, Sweden), a program based on ORACLE. Then, blood samples were centrifuged at 3,075 × *g* for 5 min. Red blood cells were deposited in the bottom of the tube and the serum was at the top. About 250 μL of each serum sample was collected in 96-wells plates, which were identified with the corresponding registration number.

A BioChek IBD ELISA kit (BioChek, ER Reeuwijk, The Netherlands) was used to detect IBD antibodies in sera. Titers were calculated as described by the manufacturer. Mean titers less than 4,000 indicate no proper vaccination without infection, mean titers between 4,000 and 14,000 suggest a proper broiler vaccination, and titers higher than 14,000 was considered as infection.

### Serologic Monitoring Tool

Results obtained were represented on a dynamic map. For this purpose, 3 main phases were developed: data collection, data analysis, and data representation.

Data collection was performed using the processes of extract, transform, and load, which allowed to obtain data from multiple different sources. Final data were loaded into another database to be analyzed in another operating system. Thus, Oracle and BioChek 2010 software were integrated for this job.

For data analysis, we developed a computer application called Online Analytical Processing (**OLAP**), allowing dynamic and geographic analysis with multidimensional cubes containing serologic response information and integrating the results of IBD of this study. A cube is a multidimensional database in which the physical storage of data is performed in a multidimensional vector. We can consider OLAP cubes as an extension of the 2 dimensions of a spreadsheet, which can be more than 3 dimensions, also called hypercubes.

Finally, after the data were obtained using the ETL processes and analyzed using the OLAP tool, the next step was to represent them geographically. A geographical information system was integrated with an open-source server called GeoServer. This tool generates Spanish geographic information, such as communities, provinces, regions, or towns. Data for each sample were associated to the identification codes of each geographic unit. This information is contained in the REGA (Registro General de Explotaciones Ganaderas - Register of livestock holdings) (https://www.mapa.gob.es/es/ganaderia/servicios/) for each farm, thus establishing the relationship between geographical information system and OLAP. Thus, the data were categorized by areas using identification with colors: yellow zones correspond with mean titers less than 4,000; green areas represent mean titers between 4,000 and 14,000, and titers higher than 14,000 correspond to active infection, been colored in red.

## Results

None of the flocks studied in this study reported clinical signs of IBD. Animals average age was 43 d with an SD of ±5.

**Figure 1:**
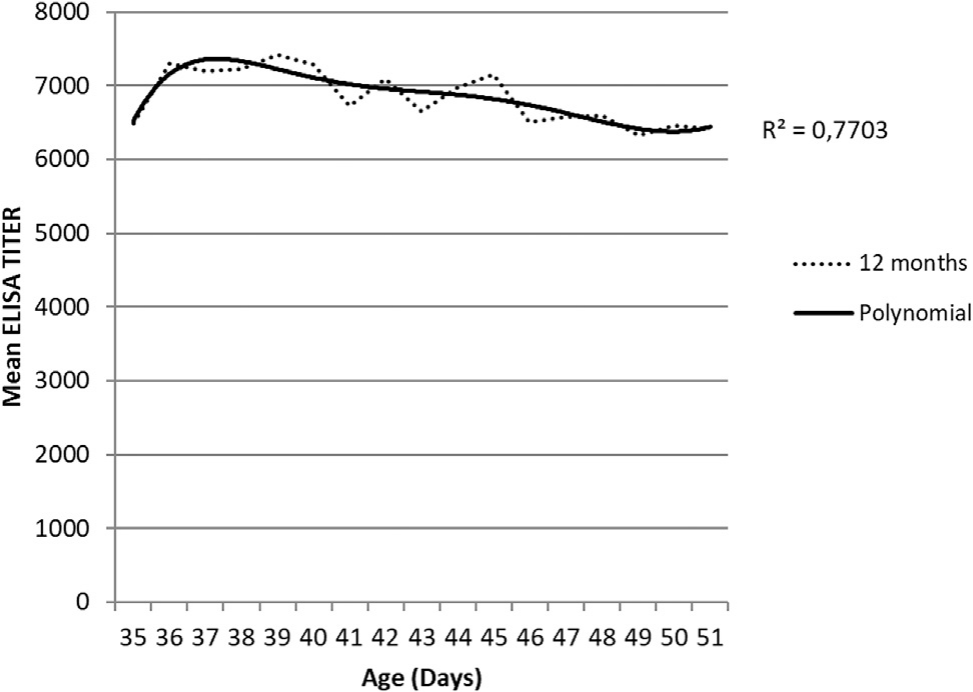
ELISA GM titers for in ovo single vaccinated broilers with an immune complex IBD vaccine during 12 mo. Abbreviation: IBD, infectious bursal disease.

From the 7,576 sera samples analyzed, the mean titer and the mean CV were 6,846 and 28.3%, respectively. Figure 1 represents the ELISA mean titer against IBD as per broiler age. The polynomial curve for mean titer fit equation: y = −0.003x^6^ + 0.2187x^5^ – 6.1473x^4^ + 84.257x^3^ – 589.46x^2^ + 1,900.8x + 5,130.1; where y is the mean titer and x the broiler age in d; R^2^ was 0.7703.

**Figure 2:**
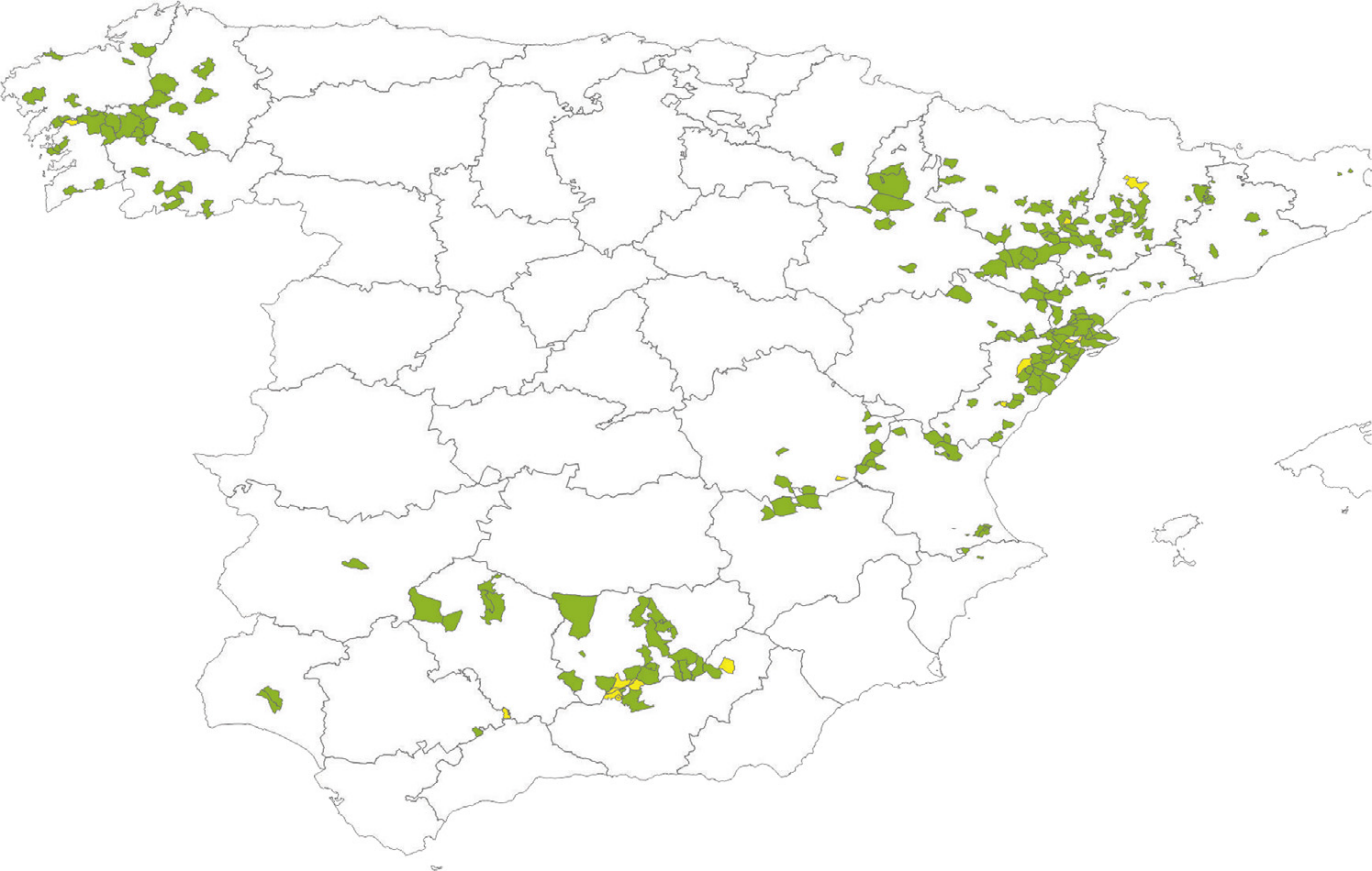
IBD serologic map throughout broiler farms in Spain during 12 mo. Abbreviation: IBD, infectious bursal disease.

Regarding serologic monitoring data, Figure 2 represents the map of Spain, which graphed the seroprevalence of virus antibodies, taking into account the spatial distribution and 12-mo period data. Titers less than 4,000 were obtained in the following regions: Altorricón, Benalúa de las Villas, Catí, Costur, La Galera, Isona i Conca Dellà, Montillana, Noalejo, Valga, and Villarta. No active infection was detected in this study.

## Discussion

Suitable vaccine monitoring at the hatchery together with and efficient administration to d-old chick or embryonated chicken egg is critical to obtain consistent and uniform immunization flock against mainly infectious poultry diseases. This fact is especially important when only a single-dose vaccine is used to protect the chickens during its lifespan (Balaguer et al., 2013).

In this study, a mapping tool was applied and assessed to control IBD immunization in 603 flock ranging from 35 to 51 d after the immunization by in ovo vaccination at 18-day-old chicken embryos. Results of this study suggested a high uniform immunization against IBDV and a protective immunization between 35 and 51 d of age, with mean titer values ranging between 6,331 and 7,426, yet, some titers lower than 4,000 were observed. This fact could be owing to unsuitable management of the vaccine administration of an immune system failure (Balaguer et al., 2013).

Moreover, the polynomial curve for mean IBD titer fit an equation with R^2^ of 0.77, suggesting a certain capacity to predict the mean titers in broilers after this vaccination for new flocks. Regarding the mapping tool, most of the areas were colored in green. This result shows the mean titers after vaccination are within expected values for vaccine humoral response, considering the absence of reported clinical signs of IBD.

The usefulness and effectiveness of epidemiologic maps has been previously to control important poultry diseases such as avian influenza, *M. gallisepticum,* or *Salmonella* (Garcia et al., 2015, 2016, 2018). The results obtained in these studies were based on seroprevalence for avian influenza and *Mycoplasma* and microbiological qualitative results for *Salmonella*, which confirms the versatility and applicability of these epidemiologic tools for disease control.

This tool help to easily monitor, in large-scale studies, the serologic response to in ovo single broiler vaccination against IBD using an immune complex IBD vaccine, including geographical and temporal information. This tool enables to compare the humoral response of broilers in different geographical locations during a period outlining the infection pressure, hatchery vaccine efficiency administration, and the immunization homogeneity.

Globalization and animal movement have led the monitoring of infectious diseases among animal farms to avoid the spread of pathogens worldwide. For this reason, mapping tools to control animal immunization is a suitable measure from the point of view of biosecurity.

The use of similar informatics tools has grown significantly in the last few yr in several animal control diseases such as bluetongue (Savini et al., 2003; Conte et al., 2005; Dall'Acqua et al., 2006; Calistri et al., 2007), shrimp epidemics (Bayot et al., 2008), and others (Savini et al., 2007). Literature has reflected the use of geographical information system technology in the prevention of avian diseases as avian influenza in Italy and China (Ehlers et al., 2003; Mannelli et al., 2006; Chang et al., 2007). However, no real-time informatics tools have been implemented since now to improve IBD humoral immunization control in broilers.

In conclusion, the results from this study suggest a suitable immunization against IBD in broiler flocks after in ovo single vaccination with an immune complex vaccine. In addition, by the means of a mathematical equation, humoral response to IBD vaccine could be predicted with a R^2^ of 0,77. Finally, this tool that incorporates newly developed mapping seroprevalence map could be an effective way for veterinary services to control and prevent diseases, such as IBD.
